# Chronic neuropathic pain components in whiplash-associated disorders correlate with metabolite concentrations in the anterior cingulate and dorsolateral prefrontal cortex: a consensus-driven MRS re-examination

**DOI:** 10.3389/fmed.2024.1404939

**Published:** 2024-07-29

**Authors:** Irene Pinilla-Fernández, Marta Ríos-León, Dinesh Kumar Deelchand, Leoncio Garrido, Mabel Torres-Llacsa, Fernando García-García, Marta Vidorreta, I. Betina Ip, Holly Bridge, Julian Taylor, Andrés Barriga-Martín

**Affiliations:** ^1^Sensorimotor Function Group, Hospital Nacional de Parapléjicos, SESCAM, Toledo, Spain; ^2^Instituto de Investigación Sanitaria de Castilla La Mancha (IDISCAM), Toledo, Spain; ^3^Grupo de Sistemas Complejos, Universidad Politécnica de Madrid, Madrid, Spain; ^4^Department of Radiology, Center for Magnetic Resonance Research, University of Minnesota, Minneapolis, MN, United States; ^5^Departamento de Química-Física, Instituto de Ciencia y Tecnología de Polímeros (ICTP-CSIC), CSIC, Madrid, Spain; ^6^Servicio de Radiodiagnóstico, Hospital Nacional de Parapléjicos, SESCAM, Toledo, Spain; ^7^Siemens Healthineers, Madrid, Spain; ^8^Wellcome Centre for Integrative Neuroimaging, FMRIB, Nuffield Department of Clinical Neurosciences, University of Oxford, Oxford, United Kingdom; ^9^Harris Manchester College, University of Oxford, Oxford, United Kingdom; ^10^Research Group in Spine Pathology, Orthopedic Surgery and Traumatology Unit, Hospital Nacional de Parapléjicos, SESCAM, Toledo, Spain; ^11^Faculty of Medicine, University of Castilla La Mancha, Toledo, Spain

**Keywords:** glutamate, n-acetyl-aspartate, choline, neuropathic pain, whiplash injury, anterior cingulate cortex, dorsolateral prefrontal cortex, occipital cortex

## Abstract

**Introduction:**

Whiplash injury (WHI) is characterised by a forced neck flexion/extension, which frequently occurs after motor vehicle collisions. Previous studies characterising differences in brain metabolite concentrations and correlations with neuropathic pain (NP) components with chronic whiplash-associated disorders (WAD) have been demonstrated in affective pain-processing areas such as the anterior cingulate cortex (ACC). However, the detection of a difference in metabolite concentrations within these cortical areas with chronic WAD pain has been elusive. In this study, single-voxel magnetic resonance spectroscopy (MRS), following the latest MRSinMRS consensus group guidelines, was performed in the anterior cingulate cortex (ACC), left dorsolateral prefrontal cortex (DLPFC), and occipital cortex (OCC) to quantify differences in metabolite concentrations in individuals with chronic WAD with or without neuropathic pain (NP) components.

**Materials and methods:**

Healthy individuals (*n* = 29) and participants with chronic WAD (*n* = 29) were screened with the Douleur Neuropathique 4 Questionnaire (DN4) and divided into groups without (WAD-noNP, *n* = 15) or with NP components (WAD-NP, *n* = 14). Metabolites were quantified with LCModel following a single session in a 3 T MRI scanner within the ACC, DLPFC, and OCC.

**Results:**

Participants with WAD-NP presented moderate pain intensity and interference compared with the WAD-noNP group. Single-voxel MRS analysis demonstrated a higher glutamate concentration in the ACC and lower total choline (tCho) in the DLPFC in the WAD-NP versus WAD-noNP group, with no intergroup metabolite difference detected in the OCC. Best fit and stepwise multiple regression revealed that the normalised ACC glutamate/total creatine (tCr) (*p* = 0.01), DLPFC n-acetyl-aspartate (NAA)/tCr (*p* = 0.001), and DLPFC tCho/tCr levels (*p* = 0.02) predicted NP components in the WAD-NP group (ACC *r*^2^ = 0.26, α = 0.81; DLPFC *r*^2^ = 0.62, α = 0.98). The normalised Glu/tCr concentration was higher in the healthy than the WAD-noNP group within the ACC (*p* < 0.05), but not in the DLPFC or OCC. Neither sex nor age affected key normalised metabolite concentrations related to WAD-NP components when compared to the WAD-noNP group.

**Discussion:**

This study demonstrates that elevated glutamate concentrations within the ACC are related to chronic WAD-NP components, while higher NAA and lower tCho metabolite levels suggest a role for increased neuronal–glial signalling and cell membrane dysfunction in individuals with chronic WAD-NP components.

## 1 Introduction

Whiplash injury (WHI) is characterised by a forced flexion extension of the neck, which frequently occurs after motor vehicle collisions, and may involve damage to intervertebral joints, discs, ligaments, muscles, and nerve roots (1). Symptoms of whiplash-associated disorders (WAD) include persistent neck pain, headache, dizziness, concentration disturbance, sleeping difficulties, and fatigue (2, 3). WAD symptoms usually resolve within 3 months, but approximately 30 and 50% of participants experience chronic pain for longer than 6 months (2–6). Although WAD is characterised by regional musculoskeletal symptoms, the development of central pathophysiological mechanisms that lead to neuropathic pain (NP) descriptors and sensory changes have also been described (3, 7–9). There is a need therefore to understand the central and peripheral pathophysiological mechanisms to improve the early diagnosis and prevention of chronic WAD symptoms, including high-impact chronic NP components (6).

Proton magnetic resonance spectroscopy (^1^H MRS) is a non-invasive technique that enables quantification of metabolite concentration and can provide an essential insight into pathophysiological mechanisms and therapeutic targets (10–14). Quantification of metabolite concentrations within brain pain-processing areas permits a mechanistic approach to detect site-specific biochemical changes in neuronal and glial cell dysfunction and their relationship with nociceptive and neuropathic pain types (10, 13, 14). Indeed, differences in key metabolite concentrations, such as glutamate (15–17), N-acetyl-aspartate (10, 18, 19), and GABA (20, 21), have been detected with pain subtypes. Furthermore, brain MRS has been used to demonstrate the therapeutic effects of analgesic treatments (22–24) and non-invasive neuromodulation of the cortex (25, 26). Metabolite concentrations within the anterior cingulate cortex (ACC) and periaqueductal grey matter (PAG) are known to correlate with WAD-NP components and endogenous pain modulation during chronic WAD, possibly related to changes in glutamatergic and neuroinflammatory mechanisms (27). However, no general difference in metabolite concentrations has been identified within the primary motor cortex, somatosensory cortex, ACC, or PAG when compared between individuals with WAD with or without chronic pain (27, 28). Furthermore, the involvement of metabolite modulation within other key areas, such as the dorsolateral prefrontal cortex (DLPFC), during chronic WAD pain has not been previously reported (29).

The technical challenges associated with ^1^H MRS acquisition methodology and metabolite analysis (30, 31) may explain the failure to detect subtle differences in metabolite concentrations related to chronic WAD pain. This limitation can be addressed with the use of the semi-adiabatic localisation by adiabatic selective refocusing sequence (semi-LASER) (30), single-voxel spectroscopy acquisition for specific anatomical regions (30, 31), and the development and implementation of a simulated basis set into the analysis (30). Analysis programmes available on scanner software are usually less sensitive, and therefore, expert consensus groups, such as the MRSinMRS group, recommend software that allows pre-processing, such as phase and frequency correction and final metabolite quantification (30). Furthermore, the inclusion of anatomical areas as reference areas to assess differences in metabolite concentrations in brain areas unrelated to specific pain types is not commonly adopted (27).

This study aimed to quantify a difference in metabolite concentration levels in the brain, in participants with chronic WHI pain screened for neuropathic components, within the ACC and the left dorsolateral prefrontal cortex (DLPFC), areas known to specifically modulate pain-related affective and mood components (32–34). The secondary aim of the study was to identify key metabolites related to chronic neuropathic pain components during chronic WHI.

## 2 Materials and methods

### 2.1 Ethics statement

This study protocol was approved by the local Clinical Research Ethics Committee (Approval number #2559/674; 2021) and was conducted at the National Hospital for Paraplegics in Toledo according to the Helsinki Declaration (35).

### 2.2 Study participant recruitment

Participants with WAD were recruited by orthopaedic surgeons at a hospital in Toledo (Spain). The period of recruitment was between September 2021 and July 2023. All individuals screened for eligibility provided written informed consent before their inclusion in the study. No *a priori* sample size calculation was made as similar studies performed in the chronic WHI phase did not detect differences in brain metabolite concentrations in individuals with WAD general pain (27, 28).

### 2.3 Inclusion criteria

For participants to be eligible, they were required to meet the following conditions: (1) clinical diagnosis of acute WAD assessed within 72 h of a traffic accident; (2) present WAD with WAD grades of between II-III according to the Quebec Task Force grading system (36–38), (3) daily pain intensity of >3 rated on the 11-point numeric rating scale (NRS) reported within 1 week of injury; (4) one or more specific descriptors assessed with the DN4 questionnaire (see below); (5) more than 3 months after WHI; and (6) 18 years of age or older (39). The exclusion criteria were as follows: (1) a history of chronic pain and/or rheumatic, neurological, or psychiatric diseases; (2) diseases causing potential neural damage (e.g., diabetes, diseases of the immune system, and oncological diseases); (3) bone injuries associated with trauma and detected in the X-ray of the cervical spine; (4) previous clinical history of cervical injuries (e.g., disc herniation, osteoarthritis, and WAD), frequent headaches, and/or orofacial pain; (5) a history of cervical surgery or surgery to the upper extremity; and (6) treatment for chronic pain previously received for long periods of time (39).

### 2.4 Assessment of pain interference

The Brief Pain Inventory (BPI) questionnaire was used to assess the patient’s perception of pain severity and its interference with several dimensions of daily life (27, 40). The pain interference scale includes pain interference related to general activity, mood, enjoyment of life, walking ability, ability to work and perform daily tasks, and relationships with other people (40, 41). BPI pain interference was calculated as a total score of the seven items (including the sleep item) and was also calculated as subscores (41) for psychological affective interference [relationships with others, enjoyment of life and mood (REM)] and physical activity interference [walking ability, general activity, and ability to work (WAW)] (41).

### 2.5 Assessment of NP components

#### 2.5.1 Physician assessment

The presence of NP components was assessed using conventional physician assessment, which was considered the gold standard (39, 42). The physician assessment was performed following routine clinical practise (39), international recommendations (43–45), and the NeuPSIG neuropathic pain criteria for probable NP (45).

This evaluation included detailed history, physical examination (e.g., movement testing, clinical bedside somatosensory function testing, and general neurological and clinical testing), and appropriate diagnostic workup including pain distribution and sensory examination (43).

#### 2.5.2 Douleur neuropathique 4 screening questionnaire

The DN4 questionnaire is a reliable tool with high discriminatory value for the identification of NP symptoms and signs (46, 47) and has proven valid for mixed pain syndromes (sensitivity: 83%; specificity: 90%) (47). The Spanish version of the DN4 with substantial inter-rater reliability (Cohen’s kappa coefficients: 0.79) and internal consistency (Cronbach’s α: 0.7) has been used (39, 46). This questionnaire consists of a total of 10 items (NP descriptors): 7 items are related to the quality of pain (burning, painful cold, and electric shocks) and its association with abnormal sensations (tingling, pins and needles, numbness, and itching), and 3 items are related to clinical examination in the painful area (touch hypoesthesia, pinprick hypoesthesia, and tactile allodynia). A score of 1 is given to each positive (yes) item. The total score is calculated as the sum of the 10 items, and the cutoff value to determine the presence of NP components is a total score of DN4 of ≥4 (46).

The presence of NP components using the DN4 questionnaire was determined according to the following characteristics of pain: (1) the presence of pain descriptions such as burning or hot, electric shocks or shooting, painful cold, pricking or pins and needles, pain evoked by light touching or loss of sensitivity to mechanical stimuli, or non-painful sensations such as numbness and tingling (46, 47) and (2) the presence of abnormal findings in the clinical examination such as the sensory change to mechanical stimuli (46, 47).

### 2.6 Brain imaging data acquisition and processing

A total of 29 healthy participants with no chronic pain and 29 participants with WHI [WAD-noNP (*n* = 15) and WAD-NP (*n* = 14)] consented to brain imaging performed with a 3 T whole body system MRI scanner (Siemens Magnetom TrioTim Syngo MR B19) with 32-channel Rx CP head coil (Siemens). MRS acquisition and analysis parameters are included as an MRSinMRS checklist (Supplementary Table S1). First, T1-weighted structural images were acquired using a three-dimensional magnetisation-prepared rapid gradient-echo (3D MPRAGE) (48) with the following parameters: 256 slices, slice thickness = 0.90 mm, TR/TE = 2300/3.01 ms, flip angle = 9°, and isotropic voxel size = 0.9 mm. The anatomical information was used for MRS voxel placement, and the images were segmented using SPM12 to determine the fractions of grey and white matter and cerebrospinal fluid volume in each region of interest (ROI) (49). The percentage grey matter for the non-injured and chronic WHI groups for each ROI was as follows: ACC:—47.9 ± 3.8% vs. 47.4 ± 4.1%, OCC: 63.4 ± 6.9% vs. 64.7 ± 3.7%, and DLPFC: 33.0 ± 6.4% vs. 32.2 ± 9.5%. These grey matter percentages facilitate discussion of neuronal or white matter differences in key metabolites detected with MRS.

Single-voxel MRS was acquired using the pulse sequence MEGA-semi-LASER SVS (CMRR Spectroscopy Package Release 2017–07, University of Minnesota: mslaser, TE = 85 ms, TR = 3,000 ms, bandwidth = 2 KHz, average of 128 scans (64 scans for edit-off and 64 for edit-on), 2,048 data points, and total scan time = 10.8 min per ROI) with a field strength of 3 T. A longer TE time was adopted to optimise GABA + quantification with the MEGA-semi-LASER sequence (50, 51). The centre frequency was −1.7 ppm, and the shimming method was achieved using the Siemens shim “Brain” application (System 3D-GRE). An unsuppressed water reference was acquired, thus the water suppression method used was VAPOR with an optional embedded outer volume suppression (OVS) to suppress water and improve the localisation of the volume of interest (VOI).

**Figure 1:**
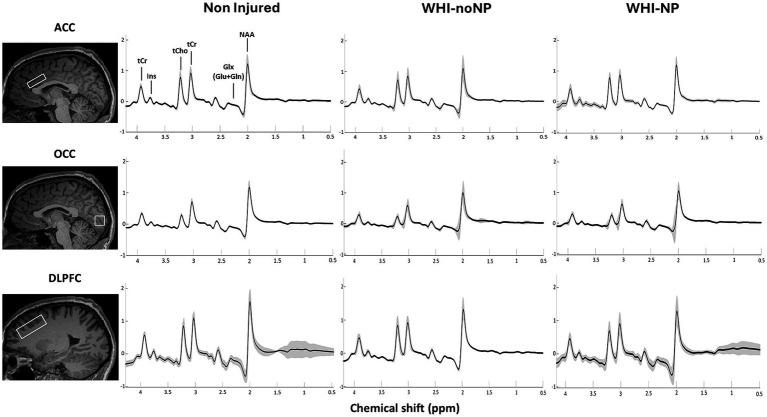
An example of a representative MR spectra, illustrating voxel mask placement for the anterior cingulate cortex **(A)**, occipital cortex **(B)**, and left dorsolateral prefrontal cortex **(C)**. Typical MRS spectra from the anterior cingulate cortex of a healthy participant **(D)**.

Voxels were positioned manually in the ROIs in the axial plane by well-trained technicians with many years of experience with a 3 T scanner for MRS under the supervision of a radiologist experienced in the identification of anatomical landmarks (52). MRS acquisition of spectra within each ROI was always in the same order. The first voxel (35 × 35 × 10 mm^3^) was placed in the anterior cingulate cortex (ACC) (Figure 1A), the second voxel (20 × 20 × 20 mm^3^) was placed in the occipital lobe (OCC) (Figure 1B), and the third voxel (20 × 20 × 40 mm^3^) was placed in the left dorsolateral prefrontal cortex (DLPFC) (Figure 1C). Voxel size was based on previous studies (26, 27). MRS voxels were first registered to the T1-anatomical space and segmented (grey matter, white matter, and CSF) using SPM12 (53, 54). In this study, spectra were acquired from the DLPFC in a smaller cohort (WAD-noNP, *n* = 13, WAD-NP *n* = 17) compared to the number of individuals with spectra obtained from the ACC and OCC (WAD-noNP *n* = 29, WAD-NP *n* = 29). During the MRS data acquisition, participants were not given specific instructions.

All MRS spectra (see Figure 1D as an example) were obtained in DICOM (.IMA) format and processed using MRspa version 1.5f (55), which runs with MATLAB R2022b (56). For GABA + quantification, MRS data were obtained as the difference between two separate measurements (64 spectra for each edit-on and edit-off). The MRspa freeware spectral processing and analysis package was used in conjunction with programs SPM12 and LCModel version 6.3-1R (for fitting and quantification of metabolites). SPM12 and LCModel interface with MRspa (53, 54, 56, 57). Frequency and phase corrections were performed followed by eddy current correction. The resulting summed semi-LASER spectra were fitted using LCModel and were scaled to water. Final GABA+ concentration was calculated from the edited spectra by calculating the difference between edit-on and edit-off spectra, while the rest of the metabolites were fitted from the edit-off spectra.

The basis set file was created specifically for our sequence MEGA-semi-LASER SVS by Dr. Deelchand from the Center for Magnetic Resonance Research at the University of Minnesota. No macromolecules were included in the basis files. The basis set contained 18 basis spectra for edit-off, out of which 7 were reported: total creatine (Cr), total n-acetyl-aspartate (NAA), inositol (Ins), total choline (Cho), glutamate and glutamine (Glx), glutamate (Glu), and gamma-aminobutyric acid (GABA+) and normalised to total creatine (tCr). Importantly, no difference in creatine levels was identified between groups, and therefore, metabolite ratio values were calculated using tCr. In this study, glutamate was not highly correlated with glutamine (Spearman’s correlation: 0.43) in line with other sequences using 3 T scanners (58). Quality control measures as recommended by the MRSinMRS consensus group are shown in Supplementary Table S2 and are presented as signal-to-noise ratio (SNR), line width (LW), full-width-half maximum (FWHW), and Cramér–Rao Lower Bound (CRLB) for each metabolite.

### 2.7 Statistical analyses

Statistical analysis was performed with a commercial software package (SigmaPlot 11.0 for Windows, Systat Software, Inc., Germany). Metabolite data were expressed either as mean ± standard deviation or as median values with 25th–75th interquartile percentiles, according to the Shapiro–Wilk normality test. Intergroup differences in metabolite concentrations were detected either with the Student’s *t*-test or Mann–Whitney test for comparisons between the healthy and WHI groups or with the one-way analysis of variance (ANOVA) or Kruskal–Wallis test for comparisons between the three groups: healthy, WAD without NP (WAD-noNP), and WAD with NP (WAD-NP). Bonferroni tests were performed with the Holm–Sidak or Dunn’s method. Intergroup clinical data were compared using the Student’s *t*-test or Mann–Whitney test. Differences and the impact of age and sex on significant differences in metabolite concentrations were assessed with an analysis of covariance (ANCOVA) using a different commercial package (JASP, version 0.18.1.0). The possible impact of age-related differences on metabolite concentrations in the healthy younger non-injured group was also controlled by performing analysis with the general WAD group and also specifically by comparing differences between the WAD-noNP and WAD-NP groups.

To reduce the number of multiple comparisons between brain MRS metabolites within the ACC, DLPFC, and OCC with the DN4 screening score, a best-fit analysis was first performed for each metabolite (SigmaPlot 11.0). A forward stepwise multiple linear regression analysis was performed to identify the best predictive model for both the metabolite concentrations for total Cr, total NAA, Ins, total Cho, Glx, Glu, and GABA and also for metabolite concentrations normalised to total Cr (27). In addition, the statistical power was reported for each predictive model.

Spearman’s correlation coefficient was calculated to assess the relationship of the metabolite concentrations that best predicted NP components as measured with the DN4 screening questionnaire.

## 3 Results

### 3.1 Demographic and clinical characteristics

Demographic and clinical data of recruited participants are presented in Table 1. No significant differences based on age or sex were seen in non-injured participants compared with those with WHI, or with the WAD-noNP-NP and WAD-NP. Furthermore, no differences were revealed between groups with reference to the time of clinical or MRS evaluation.

**Table 1:** Demographic and clinical characteristics of healthy non-injured individuals, participants with whiplash-associated disorders without neuropathic pain (WAD-noNP), and participants with whiplash-associated disorders with neuropathic pain components (WAD-NP).

	Non-injured (*n* = 29)	WAD (*n* = 29)	WAD-noNP (*n* = 15)	WAD-NP (*n* = 14)
Age	25.0 (21.8–40.8)	39.0 (30.0–45.8)**	40.0 (33.5–48.8)	38.5 (29.0–43.0)
Sex, n (%) female	16.0 (55.2%)	20 (69.0%)	8 (53.3%)	12 (85.7%)
% WAD IIa	0	3 (10.3%)	3 (20%)	0^##^
% WAD IIb	0	14 (48.3%)	10 (66.7%)	4 (28.6%)^##^
% WAD III	0	12 (41.4%)	2 (13.3%)	10 (71.4%)^##^
Evaluation time since WHI (days)	0	100 (89–108)	102 (95–108)	98 (87–117)
MRS time since WHI (days)	0	100.6 ± 32.7	94.1 ± 27.1	108.1 ± 37.9
7-day pain intensity (NRS:0–10)	0	4 (1–6)	1 (0–4)	6 (4–6) **†**
DN4 (0–10)	0	3.0 (0.8–6.0)	1.0 (0.0–3.0)	6.0 (4.0–7.0) **††**
Pain interference (BPI REM)	0	1.5 (0.0–4.8)	0.0 (0.0–0.3)	4.7 (2.9–7.3) **††**
Pain interference (BPI WAW)	0	2.7 (0.0–5.7)	0.0 (0.0–1.3)	5.7 (3.5–7.3) **††**
Pain interference (total BPI)	0	2.8 (0.2–5.6)	0.3 (0.0–1.2)	5.2 (4.1–7.3) **††**

Data are shown as mean (± SD) or median scores (25th–75th percentiles).**(Non-injured vs. WHI, *t*-test, *p* < 0.01).^##^(WAD-noNP vs. WAD-NP, chi-square, *p* < 0.01).^†^(WAD-noNP vs. WAD-NP, Mann–Whitney, *p* < 0.01).^††^(WAD-noNP vs. WAD-NP, Mann–Whitney, *p* < 0.001).DN4, Douleur Neuropathique 4; NRS, Numeric Rating Scale; BPI, Brief Pain Inventory: REM (Relation with others, Enjoyment of Life, Mood), WAW (Walking, General Activity, Working); WAD-noNP, whiplash-associated disorders without neuropathic pain; WAD-NP, whiplash-associated disorders with neuropathic pain.

Significant differences were revealed in the clinical characteristics for the number of participants diagnosed with WHI with WAD IIa, IIb, and III (*p* < 0.001). Specifically, participants reported NP components with either a WAD IIb (29%) or III (72%) grade. Finally, higher scores for 7-day pain intensity and interference were measured with the total BPI or subscores when compared between the WAD-noNP and WAD-NP groups (*p* < 0.001) (Table 1).

### 3.2 Metabolite concentrations within the OCC, ACC, and DLPFC

No difference in concentration (mM) or normalised metabolite levels were revealed in the OCC compared to the groups (Table 2). In contrast, concentration (mM) and normalised glutamate levels within the ACC were higher in the WAD-NP group compared to the WAD-noNP group. The normalised Glu/tCr concentration was higher in the healthy compared to the general WAD group (*p* < 0.01) and WAD-noNP group within the ACC (Table 3) (*p* < 0.05), but not in the DLPFC (Table 4) or OCC (Table 2). Furthermore, the tCho/tCr metabolite ratio within the DLPFC was higher in the WAD-noNP than in the healthy group (Table 4) (*p* < 0.025). Neither sex nor age or normalised metabolite concentrations affected concentration (mM) related to WAD-NP components when compared to the WAD-noNP group..

**Table 2:** Occipital cortex (OCC) metabolite concentration (mM) and metabolite ratios normalised to total creatine (tCr) in healthy non-injured individuals, participants with whiplash-associated disorders without neuropathic pain (WAD-noNP), and participants with whiplash-associated disorders with neuropathic pain components (WAD-NP).

	Non-injured (*n* = 29)	WAD (*n* = 29)	WAD-noNP (*n* = 15)	WAD-NP (*n* = 14)
tCr	5.60 ± 0.48	5.70 ± 0.53	5.64 ± 0.55	5.77 ± 0.53
tCho	1.02 ± 0.11	1.08 ± 0.16	1.06 ± 0.16	1.11 ± 0.16
NAA	10.82 ± 1.08	10.91 ± 1.3	10.77 ± 1.41	11.05 ± 1.20
Glx	7.04 (6.07–8.06)	6.88 (6.05–7.72)	6.10 ± 1.96	7.24 ± 1.41
Ins	6.45 (5.98–7.02)	7.06 (6,33–7,31)	6.94 (86.30–7.23)	7.00 (6.08–7.35)
GABA	1.34 (0.98–1.48)	1.31 (1.14–1.66) ¶ ¶	1.30 (1.09–1.51) ¶	1.60 (1.17–1.78)
Glu	2.97 (2.59–3.67)	2.74 (1.77–3.70) ¶	2.70 ± 1.39 ¶	2.68 ± 1.19
tCho/tCr	0.18 ± 0.02	0.19 ± 0.02	0.19 ± 0.02	0.19 ± 0.017
NAA/Cr	1.93 ± 0.11	1.91 ± 0.14	1.91 ± 0.15	1.92 ± 0.15
Glx/tCr	1.21 (1.12–1.39)	1.23 (1.06–1.28)	1.16 (0.97–1.28)	1.24 (1.15–1.37)
Ins/tCr	1.18 ± 0.14	1.22 ± 0.19	1.25 ± 0.22	1.19 ± 0.15
GABA/tCr	0.23 (0.19–0.26)	0.23 (0.2–0.28) ¶ ¶	0.23 (0.19–0.27) ¶	0.26 (0.20–0.32) ¶
Glu/tCr	0.54 ± 0.15	0.48 ± 0.18 ¶ ¶	0.51 ± 0.18 ¶ ¶	0.46 ± 0.19

Cr, Total creatine; NAA, total n-acetyl-aspartate; Ins, Inositol; tCho, total choline; Glx, glutamate and glutamine; Glu, glutamate; GABA+, gamma-aminobutyric acid. tCho is calculated as the sum of phosphocholine (pCho) and glycerophosphocholine (GPC).*(non-injured vs. WAD, *t*-test, *p* < 0.05).^¶^(1 subject with unanalysable metabolite data).^¶¶^(2 subjects with unanalysable metabolite data).Data are shown as mean (± SD) or median scores (25th–75th percentiles).

**Table 3:** Anterior cingulate cortex (ACC) metabolite concentration (mM) and metabolite ratios normalised to total creatine (tCr) in healthy non-injured individuals, participants with whiplash-associated disorders without neuropathic pain (WAD-noNP), and participants with whiplash-associated disorders with neuropathic pain components (WAD-NP).

	Non-injured (*n* = 29)	WAD (*n* = 29)	WAD-noNP (*n* = 15)	WAD-NP (*n* = 14)
tCr	6.58 (5.77–6.83)	6.54 (5.96–7.14)	6.54 (5.92–7.19)	6.57 (5.98–6.95)
tCho	2.16 (1.93–2.33)	2.19 (1.84–2.52)	2.32 (1.87–2.54)	2.17 (1.85–2.38)
NAA	10.26 (8.35–11.09)	10.44 (8.51–11.01)	10.44 (8.48–11.13)	10.31 (8.73–10.68)
Glx	7.64 ± 1.20	7.53 ± 1.31	7.44 ± 1.28	7.63 ± 1.38
Ins	7.15 ± 1.31	7.56 ± 1.44	7.59 ± 1.58	7.53 ± 1.34
GABA	1.12 (1.05–1.35)	1.26 (1–1.50)	1.19 ± 0.32	1.29 ± 0.32
Glu	5.15 ± 0.97	4.62 ± 1.19	4.21 ± 1.33**#1**	5.05 ± 0.85**◊#2**
tCho/tCr	0.34 (0.32–0.36)	0.35 (0.32–0.39)	0.34 (0.31–0.39)	0.34 (0.32–0.37)
NAA/Cr	1.58 ± 0.11	1.52 ± 0.12	1.51 ± 0.13	1.54 ± 0.10
Glx/tCr	1.20 ± 0.09	1.17 ± 0.12	1.14 ± 0.11	1.21 ± 0.13
Ins/tCr	1.12 (1.04–1.22)	1.14 (1.04–1.32)	1.14 (1.01–1.39)	1.15 (1.05–1.30)
GABA/tCr	0.19 ± 0.04	0.19 ± 0.04	0.18 ± 0.05	0.21 ± 0.04
Glu/tCr	0.82 (0.75–0.88)	0.76 (0.67–0.79)**††**	0.67 (0.58–0.76)**#3**	0.78 (0.76–0.85) **◘◘◘#4**

Cr, Total creatine; NAA, total n-acetyl-aspartate; Ins, Inositol; tCho, total choline; Glx, glutamate and glutamine; Glu, glutamate; GABA+, gamma-aminobutyric acid. tCho is calculated as the sum of phosphocholine (pCho) and glycerophosphocholine (GPC).Data are shown as mean (± SD) or median scores (25th –75th percentiles).^††^(non-injured vs. WHI, Mann–Whitney, *p* < 0.01).^◊^(one-way ANOVA, *p* < 0.05).#1(non-injured vs. WAD-noNP, Holm–Sidak *p* < 0.05).#2(WAD-noNP vs. WAD-NP, Holm–Sidak *p* < 0.05).◘◘◘(Kruskal–Wallis, *p* < 0.001).#3(non-injured vs. WAD-noNP, Dunn’s method *p* < 0.05).#4(WAD-NP vs. WAD-noNP, Dunn’s method *p* < 0.05).

**Table 4:** Left dorsolateral prefrontal cortex (DLPFC) metabolite concentration (mM) and metabolite ratios normalised to total creatine (tCr) in healthy non-injured individuals, participants with whiplash-associated disorders without neuropathic pain (WAD-noNP), and participants with whiplash-associated disorders with neuropathic pain components (WAD-NP).

	Non-injured (*n* = 13)	WAD (*n* = 17)	WAD-noNP (*n* = 8)	WAD-NP (*n* = 9)
tCr	7.65 ± 0.57	7.52 ± 0.89	7.87 ± 0.71	7.21 ± 0.96
tCho	2.48 ± 0.40	2.70 ± 0.60	3.07 ± 0.48**#1**	2.37 ± 0.51**#2**
NAA	13.43 ± 1.97	12.67 ± 1.69	12.91 ± 1.38	12.44 ± 1.99
Glx	9.75 ± 1.31	10.11 ± 1.41	10.22 ± 1.75	10.01 ± 1.14
Ins	11.18 ± 1.08	11.35 ± 2.21	12.54 (12.09–13.82)	10.98 (9.29–11.18)**◘◘#3**
GABA	1.39 ± 0.41	1.23 ± 0.38	1.22 ± 0.34	1.24 ± 0.44
Glu	5.72 ± 0.72	5.07 ± 1.48¶	5.05 ± 1.80	5.08 ± 1.19 **¶**
tCho/tCr	0.32 ± 0.05	0.36 ± 0.055	0.39 ± 0.04**#4**	0.33 ± 0.05◊◊**#5**
NAA/Cr	1.75 ± 0.19	1.69 ± 0.16	1.65 ± 0.14	1.73 ± 0.17
Glx/tCr	1.28 ± 0.14	1.35 ± 0.16	1.30 ± 0.19	1.40 ± 0.12
Ins/tCr	1.46 ± 0.13	1.50 ± 0.19	1.58 ± 0.25	1.43 ± 0.07
GABA/tCr	0.18 ± 0.05	0.17 ± 0.06	0.16 ± 0.05	0.17 ± 0.07
Glu/tCr	0.75 ± 0.09	0.68 ± 0.18¶	0.63 (0.50–0.69)	0.77 (0.70–0.82) **◘ ¶**

Cr, Total creatine; NAA, total n-acetyl-aspartate; Ins, Inositol; tCho, total choline; Glx, glutamate and glutamine; Glu, glutamate; GABA+, gamma-aminobutyric acid. tCho is calculated as the sum of phosphocholine (pCho) and glycerophosphocholine (GPC).Data are shown as mean (± SD) or median scores (25th–75th percentiles).¶(1 subject with unanalysable metabolite data).◊◊(one-way ANOVA, *p* < 0.01).#1(non-injured vs. WAD-noNP, Holm–Sidak *p* < 0.05).#2(WAD-noNP vs. WAD-NP, Holm–Sidak *p* < 0.025).◘(Kruskal–Wallis, *p* = 0.06).◘◘(Kruskal–Wallis, *p* < 0.01).#3(WAD-NP vs. WAD-noNP, Dunn’s method *p* < 0.05).#4(non-injured vs. WAD-noNP, Holm–Sidak *p* < 0.025).#5(WAD-noNP vs. WAD-NP, Holm–Sidak *p* < 0.05).

Regarding the left DLPFC, lower concentration (mM) and normalised tCho were found in the WAD-NP group than in the WAD-noNP group. Finally, a reduction in concentration (mM) Ins metabolite levels in DLPFC is shown in the WAD-NP group compared to the WAD-noNP group.

### 3.3 Best fit and forward stepwise regression analysis of MRS metabolites

**Figure 2:**
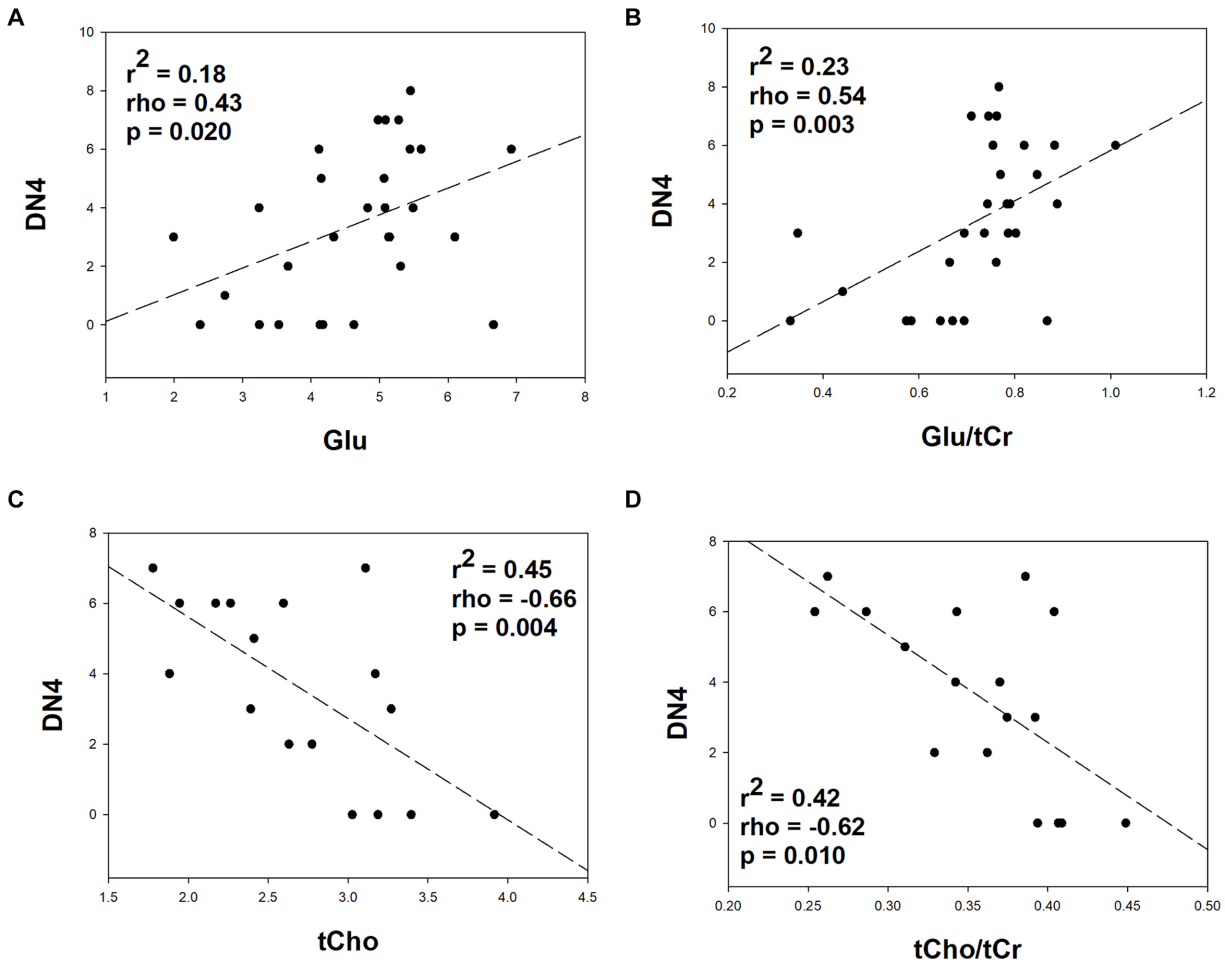
Relationship between metabolite concentrations quantified using brain MRS within the anterior cingulate (**A,B**; *n* = 29) and left prefrontal dorsolateral cortex (**C,D**; *n* = 17) and neuropathic pain components as screened with the “douleur neuropathique 4” (DN4) screening tool in participants with chronic whiplash injury. The regression coefficient, Spearman correlation, and statistical significance of the correlation are included in each graph. To reduce type II error Spearman correlations were calculated only from the best predictive models of metabolite measures for chronic WAD-NP components.

The best fit and multiple linear regression between metabolite concentrations within the OCC, ACC, and DLPFC with the DN4 screening scores is shown in Table 5. Forward stepwise regression revealed that chronic normalised glutamate concentrations predicted chronic WAD-NP components (*r*^2^ = 0.26, *p* < 0.01, alpha = 0.81) as shown in Figure 2A (rho = 0.54, *p* = 0.003). In the left DLPFC, both concentration (mM) and normalised tCho and NAA metabolite concentrations predicted chronic WAD-NP components (*r*^2^ = 0.62, with significance ranging between *p* < 0.05 and *p* < 0.001, alpha = 0.98) as shown in Figure 2B (rho = −0.66, *p* = 0.004), Figure 2C (rho = −0.62, *p* = 0.01), and Figure 2D (rho = 0.25, *p* = 0.336).

**Table 5:** Best-fit factor and forward multiple regression analysis for occipital cortex (OCC), anterior cingulate cortex (ACC), dorsolateral prefrontal cortex (DLPFC) MRS metabolite concentration (mM) and ratio normalised to tCr with DN4 scores for participants with chronic WAD with NP components.

Concentration (mM) metabolite	OCCIP	ACC	DLPFC
tCr	E.	−1.45 ± 0.69*	E.
Glx	1.23 ± 0.48*	E.	0.9 ± 0.31*
NAA	−0.65 ± 0.56	E.	0.76 ± 0.04*
tCho	E.	E.	−4.08 ± 0.89***
Ins	−0.35 ± 0.44	0.18 ± 0.35	−0.55 ± 0.03*
GABA	E.	2.48 ± 1.52	−1.02 ± 0.99
Glu	−0.68 ± 0.48	1.29 ± 0.44**	E.
Best subset regression r^2^	0.31	0.34	0.83
Forward stepwise r^2^	E.	Glu (0.91 ± 0.38) **r** ^**2**^ **= 0.18**	NAA (0.56 ± 0.34) tCho (−1.04 ± 0.95) **r**^**2**^ **= 0.62**
Power α	E.	0.63	**0.98**
Metabolite Normalised Ratio (tCr)	OCCIP	ACC	DLPFC
Glx/tCr	7.34 ± 2.96*	−0.63 ± 4.23	6.44 ± 2.34*
NAA/Cr	−3.99 ± 3.90	0.63 ± 3.78	3.09 ± 2.60
tCho/tCr	E.	E.	−30.19 ± 6.59***
Ins/tCr	E.	E.	−5.26 ± 2.14*
GABA/tCr	E.	16.74 ± 10.98	E.
Glu/tCr	−4.56 ± 2.84	8.55 ± 3.29*	E.
Best subset regression r^2^	E.	0.33	0.81
Forward stepwise r^2^	E.	Glu/tCr (8.62 ± 2.83) **r**^**2**^ **= 0.26**	tCho/tCr (−34.82 ± 8.30) NAA/Cr (7.36 ± 2.88) **r**^**2**^ **= 0.62**
Power α	E.	**0.81**	**0.98**

Cr, Total creatine; NAA, total n-acetyl-aspartate; Ins, Inositol; tCho, total choline; Glx, glutamate and glutamine; Glu, glutamate; GABA+, gamma-aminobutyric acid. tCho is calculated as the sum of phosphocholine (pCho) and glycerophosphocholine (GPC). Data are shown as mean (± SE). E. Excluded.*(*p* < 0.05), ** (*p* < 0.001), *** (*p* < 0.001). Significant results presented in bold.

## 4 Discussion

This is the first study to show significant differences in metabolite concentrations within pain-processing cortical areas, such as the ACC and DLPFC, in participants with chronic whiplash injury and WAD neuropathic pain components when compared to individuals without WAD-NP components. Importantly, the adoption of the latest MRSinMRS recommendations for metabolite analysis, including calibration with a simulated basis set into the analysis routine, and rigorous signal preprocessing including quality control, may have contributed to improved metabolite detection and quantification of metabolite differences during chronic WAD-NP. Indeed, elevated glutamate concentrations within the ACC predicted chronic WAD-NP components, while higher NAA and lower tCho metabolite levels within the DLPFC suggest a role for increased neuronal–glial signalling and neuronal membrane dysfunction with central chronic pain mechanisms. Radiological evidence for biochemical differences in affective pain-processing areas provides further evidence of the involvement of definite NP components in chronic WAD.

Although this study was performed on a small cohort of individuals reporting chronic WAD pain with neuropathic components, statistically powered predictive correlations were attained between the glutamate/tCr ratio in the ACC (α = 0.81) and the tCho/tCr and NAA/tCR ratios in DLPFC with WAD-NP components (DN4, α = 0.98). Furthermore, metabolites within the DLPFC region revealed a higher regression value with NP components in chronic WAD (*r*^2^ = 0.62 [compared to an r^2^ of 0.26 within the ACC]). Importantly stepwise regression was not able to detect predictive relationships between metabolite levels and WAD-NP components within the occipital cortex, which was included in this study as a putative control ROI.

### 4.1 Neuropathic pain components associated with chronic WAD

Approximately 34% (25–75%) of patients present NP characteristics following WHI, characterised by sensory dysfunction and nerve mechanosensitivity (59). Pain associated with WAD has often been diagnosed as musculoskeletal pain in the absence of clear evidence of nerve or brain lesions tested using neurophysiological or radiological techniques; pain and sensory disturbances in a neuroanatomically defined area consistent with a specific nerve lesion have not been forthcoming for WAD (45). However, an early study of NP symptoms and signs in patients with acute WHI (8) and, more recently, small fibre structural and functional deficits in chronic WAD have been demonstrated (9).

In this study, 48% of the chronic WHI cohort presented NP symptoms and signs as measured with the DN4 questionnaire. Compared with the WAD-noNP group, individuals with chronic WAD-NP were characterised by a higher 7-day pain intensity (NRS) and pain interference scores (BPI). For those subjects diagnosed with WAD grade II and III scores, NP symptoms and signs were detected, suggesting that NP components were associated with or without presumed central or peripheral nerve injury. The difficulty in diagnosing and differentiating musculoskeletal and NP components may lead to poor treatment, which could be improved with the extensive use of NP screening tools and the detection of pain types (39, 60). Accurate small nerve fibre examination (9) and radiological evidence of either structural or biochemical differences within pain-processing areas of the brain, especially those related to affective pain, may help to support the clinical diagnosis of chronic NP with sensory descriptors and changes during chronic WHI.

### 4.2 Current application of MRI techniques for chronic pain and consensus-driven MRS analysis

Proton magnetic resonance spectroscopy (^1^H MRS) imaging of pain-processing areas permits a mechanistic approach to detect site specific biochemical changes in neuronal and glial cell dysfunction and can be used to demonstrate the therapeutic effects of analgesic treatments (22–24) and non-invasive neuromodulation of the cortex (25, 26). However, ^1^H MRS imaging is only one of several non-invasive techniques that enable quantification of structural, functional, or biochemical alterations in brain function for chronic pain research (61) that collectively can provide an essential insight into pathophysiological mechanisms and therapeutic targets. These techniques have proven highly effective in revealing alterations in brain regions implicated in pain modulation (62, 63) and emotional processing (64, 65). However, challenges persist regarding the application of MRI techniques in pain research, such as issues related to protocol standardisation and variability in imaging results across studies. Future directions for researchers may involve refining imaging protocols to enhance reproducibility (66), adoption of multimodal imaging techniques, and developing machine learning algorithms for more precise analysis of MRI data (67–69).

The technical challenges associated with the ^1^H MRS imaging and metabolite analysis (30, 31, 70) may explain the failure to detect subtle differences in metabolite concentrations related to chronic WHI pain (27). Analysis methods available on scanner software are usually inferior to demonstrating differences in metabolite concentrations with MRS techniques, and therefore, expert consensus groups recommend the use of software that allows pre-processing, such as phase and frequency correction, quality control, tissue segmentation, and final metabolite quantification using modelling algorithms (30). In this study, the use of the semi-adiabatic localisation by adiabatic selective refocusing (semi-LASER) sequence (30), single-voxel spectroscopy imaging of specific anatomical regions (30, 31), and the development and implementation of a simulated basis set into the analysis routine (30) were implemented into the analysis routine. Furthermore, a binary mask of voxel locations was co-registered with the T1-weighted images, and this mask was applied using SPM12 scripts to determine total tissue (grey and white matter) and cerebrospinal fluid fractions in each voxel (26). A checklist for full reporting of MRS parameters in line with the recommendation of the MRSinMRS consensus group (66) was followed, and metabolite concentrations were presented as concentration (mM) values and ratios normalised to total creatine levels (71). Finally, the possible contribution of demographic cofactors, such as sex and age, should also addressed with ANCOVA (72, 73). In the present study, these techniques have ensured high-quality analysis of metabolite concentration levels and detection of group differences in the ACC and DLPFC, but not the OCC, for metabolites that predict WAD-NP components.

### 4.3 Higher glutamate concentration in ACC predicts chronic WAD-NP components

The anterior cingulate cortex (ACC) plays a leading role in chronic pain, specifically in the modulation of affective and mood components (32, 33). This is supported by synaptic, molecular structural changes in the ACC, which contribute to chronic pain states (74–76). The ACC is also implicated in the cognitive impairment in chronic pain patients, potentially mediating the impact of pain-related distress on cognitive functions (32, 77). Thus, the ACC is a potential target for neuromodulation and clinical pain management (24, 25, 78).

In this study, MRS revealed elevated concentration (mM) and normalised glutamate concentrations in the group with WAD-NP components and a significant predictive relationship between normalised glutamate ratios with the DN4 NP scores. Glutamate as the main excitatory neurotransmitter has a leading role in nociception and central sensitisation, which is associated with chronic pain (79). Glx (glutamate plus glutamine) levels pooled across pain-related brain regions have been positively associated with pain sensitivity (15), while tonic noxious stimulation leads to increased concentrations of glutamate and Glx at the onset of pain (80). Higher glutamate levels within the ACC have been shown in individuals with chronic low back pain (81, 82), while Glx levels have also been linked to psychological state and depression (82). Furthermore, treatment with transcranial direct current stimulation or morphine decreases Glx levels in the ACC (23–25), suggesting a potential mechanism for its analgesic effect. Future studies should assess the relationship between normalised ACC glutamate metabolite concentrations with pain intensity and affective measures in people with chronic WAD-NP components.

### 4.4 Lower total choline levels and higher n-acetyl-aspartate DLPFC metabolite concentrations predict the presence of chronic WAD-NP components

The dorsolateral prefrontal cortex (DLPFC) undergoes a functional and structural reorganisation in chronic pain conditions (83) and plays a crucial role in the modulation of pain perception and processing. Thus, the DLPFC exerts active control on pain perception by modulating pain signals in the brain (34). In fact, Ong et al. (84) and Loggia et al. (85) highlight the involvement of the DLPFC in pain modulation, emphasising its role in chronic pain and negative cognition-induced hyperalgesia. These findings highlight the importance of the DLPFC in chronic pain and its potential as a target for therapeutic interventions.

In this study, lower total choline (tCho) concentrations predicted WAD-NP components during chronic pain. Phosphocholine and glycerophosphoryl choline are key components in cell membrane synthesis and a marker of cellular turnover (86) and are associated with glia cells within the brain (87). Neuropathic pain can lead to changes in choline levels following head trauma within pain-processing centres (13, 19). Moreover, neuropathic pain has been associated with neuroinflammation, characterised by elevated choline-containing compounds, such as phosphocholine, found in higher concentrations in glial cells than neurons (10). However, only one study has shown reduced chronic choline levels in cases of human immunodeficiency virus infection, with initially elevated levels of choline levels which then decreased significantly at a later stage (88). Although the exact pathophysiological mechanism that implicates reduced choline levels with chronic pain is unknown, it is of interest that cortical targeting of central cholinomimetics has been suggested as an effective therapy for neuropathic pain (89). Indeed, choline supplementation can have beneficial effects on brain health, including reducing inflammation and cognitive deficits in an experimental model of Alzheimer’s disease (90).

Even though N-acetyl-aspartate is a metabolite related to neuronal density activity or cell death (91), higher concentrations of NAA have been recently identified in myelin and oligodendrocytes compared with neurons (92). Although previous studies of lower NAA levels in the DLPFC have been related to chronic back pain (12, 93), higher NAA levels have been associated in other pain-processing areas with chronic NP severity, post-traumatic stress disorder, and post-concussive symptoms in individuals with traumatic brain injury (19). Taken together, lower choline and higher NAA levels may be related to pathophysiological mechanisms associated with neuroinflammation during chronic WHI pain and may represent robust biomarkers of chronic WAD pain with neuropathic components.

### 4.5 Study limitations

This study was not conducted on age- or gender-matched individuals within each group. Importantly, analysis of covariance demonstrated that neither sex nor age or normalised metabolite concentrations affected concentration (mM) related to WAD-NP components when compared to the WAD-noNP group. However, in this study, higher glutamate concentrations were found in the healthy non-injured group than in the WHI-noNP group. In line with the demographic data presented in Table 1, which shows that the median age of the WAD-NP was 14 years lower than the general WHI group, glutamate levels are known to be higher in younger subjects (94). When metabolite concentrations in the WHI-NP group were specifically compared to the WHI-noNP control group, ROI-specific changes in glutamate were seen in both the ACC and DLPFC. Importantly, no differences in glutamate ratio concentration were observed between the non-injured and WHI-noNP control groups (Table 4). Finally, caution should also be made with the interpretation of tCho in spectroscopy studies, as higher tCho metabolite concentrations have been observed in older healthy subjects (95), which may explain the higher levels of this metabolite identified in the DLPFC for the WHI-noNP control group (Table 4).

Reliance on stepwise regression analysis to identify target metabolites associated with WAD-NP components may be influenced by several statistical problems with this test, including overfitting of data, biased estimates, and inflated type I errors (96). As such, significant best-fit metabolites should also be considered as predictors for NP components in chronic WAD components, including GABA and Ins (10, 20, 27, 97). The best-fit analysis performed in this study demonstrated that lower Ins concentrations in DLPFC are a predictor of chronic NP components, although previous studies have revealed a relationship between higher Ins levels and chronic pain with other pathologies (19, 82). The relationship between Ins metabolite levels and chronic pain may reflect differences between different pain pathologies (10, 13). In future studies, parallel adoption of multiple regression and machine learning analysis techniques may provide a better interpretation of key metabolite levels in the development of chronic pain (67, 68).

Although no metabolite predictors of chronic WAD pain were identified in the OCC, the inclusion of control areas should be more closely examined in longitudinal studies where the contribution of metabolite changes to the development of chronic pain can be assessed during acute WAD. Indeed, chronic WAD dysfunction of the visual system is associated with functional impairment in occipital cortical areas sensitive to coherent motion (98) while EEG changes in OCC have been associated with motor-evoked jaw pain (99).

Finally, total creatine concentrations in MRS studies have been used to standardise metabolite levels in the brain. In this study, no difference in creatine levels was identified between groups, although lower concentrations (mM) of creatine levels within the ACC were associated with NP components in the best-fit analysis. It is important to understand therefore that these basal Cr levels, which are expressed predominantly in glia, may also change during neuroinflammation (10, 100) or with age (73). These findings suggest that caution should be made with the normalisation of metabolite levels using ratio measures (27).

## 5 Conclusion

The results of this study show that elevated glutamate concentrations within the ACC predict chronic WAD-NP components, while higher NAA and lower total choline (tCho) metabolite levels within the DLPFC suggest a role for increased neuronal–glial signalling and cell membrane dysfunction with central chronic pain mechanisms. No chronic differences were seen in the occipital cortex, which supports the role of altered metabolite concentrations within the affective pain-processing areas such as the ACC and DLPFC. Detection of metabolite signals that reflect pathophysiological mechanisms of glutamatergic, neuroinflammatory, and cell signalling dysfunction could lead to a better understanding of the development of pathophysiological mechanisms of chronic pain that lead to high-impact chronic NP components of chronic WAD and future therapeutic targets for the neuromodulation of chronic WHI pain.

## Data availability statement

The raw data supporting the conclusions of this article will be made available by the authors, without undue reservation.

## Ethics statement

The studies involving humans were approved by the Toledo Hospital Complex Clinical Research Ethics Committee (Nº 2559/674; 17/02/2021). The studies were conducted in accordance with the local legislation and institutional requirements. The participants provided their written informed consent to participate in this study.

## Author contributions

IP-F: Writing – original draft, Writing – review & editing. MR-L: Writing – original draft, Writing – review & editing. DD: Writing – original draft, Writing – review & editing. LG: Writing – original draft, Writing – review & editing. MT-L: Writing – original draft, Writing – review & editing. FG-G: Writing – original draft, Writing – review & editing. MV: Writing – original draft, Writing – review & editing. II: Writing – original draft, Writing – review & editing. HB: Writing – original draft, Writing – review & editing. JT: Writing – original draft, Writing – review & editing. AB-M: Writing – original draft, Writing – review & editing.

## References

[1] Kasch H Qerama E Kongsted A Bach FW Bendix T Jensen TS. Deep muscle pain, tender points and recovery in acute whiplash patients: a 1-year follow-up study. Pain. (2008) 140:65–73. doi: 10.1016/j.pain.2008.07.008 18768261

[2] Daenen L Nijs J Roussel N Wouters K Van Loo M Cras P. Dysfunctional pain inhibition in patients with chronic whiplash-associated disorders: an experimental study. Clin Rheumatol. (2013) 32:23–31. doi: 10.1007/s10067-012-2085-2, PMID: 22983264

[3] Vallez Garcia D Doorduin J Willemsen AT Dierckx RA Otte A. Altered regional cerebral blood flow in chronic whiplash associated disorders. EBioMedicine. (2016) 10:249–57. doi: 10.1016/j.ebiom.2016.07.008, PMID: 27444853 PMC5006659

[4] Seroussi R Singh V Fry A. Chronic whiplash pain. Phys Med Rehabil Clin N Am. (2015) 26:359–73. doi: 10.1016/j.pmr.2015.01.003 25952070

[5] Van Oosterwijck J Nijs J Meeus M Paul L. Evidence for central sensitization in chronic whiplash: a systematic literature review. Eur J Pain. (2013) 17:299–312. doi: 10.1002/j.1532-2149.2012.00193.x, PMID: 23008191

[6] Bellosta-Lopez P Domenech-Garcia V Ortiz-Lucas M Lluch-Girbes E Herrero P Sterling M. Longitudinal changes and associations between quantitative sensory testing and psychological factors in whiplash-associated disorders: a systematic review and Meta-analyses-based data synthesis. J Pain. (2024) 25:12–30. doi: 10.1016/j.jpain.2023.07.021 37517451

[7] Davis CG. Mechanisms of chronic pain from whiplash injury. J Forensic Leg Med. (2013) 20:74–85. doi: 10.1016/j.jflm.2012.05.004 23357391

[8] Sterling M Pedler A. A neuropathic pain component is common in acute whiplash and associated with a more complex clinical presentation. Man Ther. (2009) 14:173–9. doi: 10.1016/j.math.2008.01.009 18358761

[9] Farrell SF Sterling M Irving-Rodgers H Schmid AB. Small fibre pathology in chronic whiplash-associated disorder: a cross-sectional study. Eur J Pain. (2020) 24:1045–57. doi: 10.1002/ejp.1549, PMID: 32096260

[10] Chang L Munsaka SM Kraft-Terry S Ernst T. Magnetic resonance spectroscopy to assess Neuroinflammation and neuropathic pain. J Neuroimmune Pharmacol. (2013) 8:576–93. doi: 10.1007/s11481-013-9460-x, PMID: 23666436 PMC3698315

[11] Schmidt-Wilcke T. Neuroimaging of chronic pain. Best Pract Res Clin Rheumatol. (2015) 29:29–41. doi: 10.1016/j.berh.2015.04.030 26266997

[12] Zhao X Xu M Jorgenson K Kong J. Neurochemical changes in patients with chronic low Back pain detected by proton magnetic resonance spectroscopy: a systematic review. Neuroimage Clin. (2017) 13:33–8. doi: 10.1016/j.nicl.2016.11.006, PMID: 27920977 PMC5126149

[13] Widerstrom-Noga E Pattany PM Cruz-Almeida Y Felix ER Perez S Cardenas DD. Metabolite concentrations in the anterior cingulate cortex predict high neuropathic pain impact after spinal cord injury. Pain. (2013) 154:204–12. doi: 10.1016/j.pain.2012.07.022, PMID: 23141478 PMC3670594

[14] Widerstrom-Noga E Cruz-Almeida Y Felix ER Pattany PM. Somatosensory phenotype is associated with thalamic metabolites and pain intensity after spinal cord injury. Pain. (2015) 156:166–74. doi: 10.1016/j.pain.0000000000000019, PMID: 25599312 PMC4423177

[15] Zunhammer M Schweizer LM Witte V Harris RE Bingel U Schmidt-Wilcke T. Combined glutamate and glutamine levels in pain-processing brain regions are associated with individual pain sensitivity. Pain. (2016) 157:2248–56. doi: 10.1097/j.pain.0000000000000634, PMID: 27649042

[16] Wang W Zhang X Bai X Zhang Y Yuan Z Tang H. Gamma-aminobutyric acid and glutamate/glutamine levels in the dentate nucleus and periaqueductal gray with episodic and chronic migraine: a proton magnetic resonance spectroscopy study. J Headache Pain. (2022) 23:83. doi: 10.1186/s10194-022-01452-6, PMID: 35840907 PMC9287958

[17] Pigott T McPeak A de Chastelain A DeMayo MM Rasic N Rayner L. Changes in brain Gaba and glutamate and improvements in physical functioning following intensive pain rehabilitation in youth with chronic pain. J Pain. (2023) 24:1288–97. doi: 10.1016/j.jpain.2023.02.027 36966034

[18] Moffett JR Ross B Arun P Madhavarao CN Namboodiri AM. N-Acetylaspartate in the Cns: from Neurodiagnostics to neurobiology. Prog Neurobiol. (2007) 81:89–131. doi: 10.1016/j.pneurobio.2006.12.003, PMID: 17275978 PMC1919520

[19] Robayo LE Govind V Salan T Cherup NP Sheriff S Maudsley AA. Neurometabolite alterations in traumatic brain injury and associations with chronic pain. Front Neurosci. (2023) 17:1125128. doi: 10.3389/fnins.2023.1125128, PMID: 36908781 PMC9997848

[20] Puts NA Edden RA. *In vivo* magnetic resonance spectroscopy of Gaba: a methodological review. Prog Nucl Magn Reson Spectrosc. (2012) 60:29–41. doi: 10.1016/j.pnmrs.2011.06.001, PMID: 22293397 PMC3383792

[21] Bridge H Stagg CJ Near J Lau CI Zisner A Cader MZ. Altered neurochemical coupling in the occipital cortex in migraine with visual Aura. Cephalalgia. (2015) 35:1025–30. doi: 10.1177/0333102414566860, PMID: 25631169

[22] Shen J Rothman DL. Magnetic resonance spectroscopic approaches to studying neuronal: glial interactions. Biol Psychiatry. (2002) 52:694–700. doi: 10.1016/s0006-3223(02)01502-0 12372659

[23] Hansen TM Olesen AE Simonsen CW Fischer IW Lelic D Drewes AM. Acute metabolic changes associated with analgesic drugs: an Mr spectroscopy study. J Neuroimaging. (2016) 26:545–51. doi: 10.1111/jon.12345, PMID: 27028269

[24] Hansen TM Olesen AE Simonsen CW Drewes AM Frokjaer JB. Cingulate metabolites during pain and morphine treatment as assessed by magnetic resonance spectroscopy. J Pain Res. (2014) 7:269–76. doi: 10.2147/JPR.S61193, PMID: 24899823 PMC4038455

[25] Auvichayapat P Keeratitanont K Janyachareon T Auvichayapat N. The effects of transcranial direct current stimulation on metabolite changes at the anterior cingulate cortex in neuropathic pain: a pilot study. J Pain Res. (2018) 11:2301–9. doi: 10.2147/JPR.S172920, PMID: 30349356 PMC6188066

[26] Moxon-Emre I Daskalakis ZJ Blumberger DM Croarkin PE Lyon RE Forde NJ. Modulation of dorsolateral prefrontal cortex glutamate/glutamine levels following repetitive transcranial magnetic stimulation in young adults with autism. Front Neurosci. (2021) 15:711542. doi: 10.3389/fnins.2021.711542, PMID: 34690671 PMC8527173

[27] Serrano-Munoz D Galan-Arriero I Avila-Martin G Gomez-Soriano J Florensa J Garcia-Peris A. Deficient inhibitory endogenous pain modulation correlates with periaqueductal gray matter metabolites during chronic whiplash injury. Clin J Pain. (2019) 35:668–77. doi: 10.1097/AJP.0000000000000722, PMID: 31149933

[28] Farrell SF Cowin GJ Pedler A Durbridge G de Zoete RMJ Sterling M. Magnetic resonance spectroscopy assessment of brain metabolite concentrations in individuals with chronic whiplash-associated disorder: a cross-sectional study. Clin J Pain. (2021) 37:28–37. doi: 10.1097/AJP.0000000000000890, PMID: 33093341

[29] Murillo C Lopez-Sola M Cagnie B Sunol M Smeets R Coppieters I. Gray matter adaptations to chronic pain in people with whiplash-associated disorders are partially reversed after treatment: a voxel-based morphometry study. J Pain. (2024) 25:104471. doi: 10.1016/j.jpain.2024.01.336, PMID: 38232862

[30] Wilson M Andronesi O Barker PB Bartha R Bizzi A Bolan PJ. Methodological consensus on clinical proton Mrs of the brain: review and recommendations. Magn Reson Med. (2019) 82:527–50. doi: 10.1002/mrm.27742, PMID: 30919510 PMC7179569

[31] Near J Harris AD Juchem C Kreis R Marjanska M Oz G. Preprocessing, analysis and quantification in single-voxel magnetic resonance spectroscopy: experts' consensus recommendations. NMR Biomed. (2021) 34:e4257. doi: 10.1002/nbm.4257 32084297 PMC7442593

[32] Barthas F Sellmeijer J Hugel S Waltisperger E Barrot M Yalcin I. The anterior cingulate cortex is a critical hub for pain-induced depression. Biol Psychiatry. (2015) 77:236–45. doi: 10.1016/j.biopsych.2014.08.004, PMID: 25433903

[33] Murillo C Coppieters I Cagnie B Bernaers L Bontinck J Meeus M. Neural processing of pain-related distress to neck-specific movements in people with chronic whiplash-associated disorders. Pain. (2023) 164:1954–64. doi: 10.1097/j.pain.0000000000002890, PMID: 36943244

[34] Lorenz J Minoshima S Casey KL. Keeping pain out of mind: the role of the dorsolateral prefrontal cortex in pain modulation. Brain. (2003) 126:1079–91. doi: 10.1093/brain/awg102, PMID: 12690048

[35] World Medical A. World medical association declaration of Helsinki: ethical principles for medical research involving human subjects. JAMA. (2013) 310:2191–4. doi: 10.1001/jama.2013.281053 24141714

[36] Hartling L Brison RJ Ardern C Pickett W. Prognostic value of the Quebec classification of whiplash-associated disorders. Spine. (2001) 26:36–41. doi: 10.1097/00007632-200101010-00008, PMID: 11148643

[37] Spitzer WO Skovron ML Salmi LR Cassidy JD Duranceau J Suissa S. Scientific monograph of the Quebec task force on whiplash-associated disorders: redefining "whiplash" and its management. Spine (Phila Pa 1976). (1995) 20:1S–73S. PMID: 7604354

[38] Shergill Y Cote P Shearer H Wong JJ Stupar M Tibbles A. Inter-rater reliability of the Quebec task force classification system for recent-onset whiplash associated disorders. J Can Chiropr Assoc. (2021) 65:186–92. PMID: 34658390 PMC8480375

[39] Rios-Leon M Taylor J Segura-Fragoso A Barriga-Martin A. Usefulness of the Dn4, S-Lanss and Paindetect screening questionnaires to detect the neuropathic pain components in people with acute whiplash-associated disorders: a cross-sectional study. Pain Med. (2023) 25:344–51. doi: 10.1093/pm/pnad165, PMID: 38150190 PMC11063748

[40] de Andres AJ Cruces Prado LM Canos Verdecho MA Penide Villanueva L Del Valle HM Herdman M. Validation of the short form of the brief pain inventory (bpi-sf) in Spanish patients with non-Cancer-related pain. Pain Pract. (2015) 15:643–53. doi: 10.1111/papr.12219, PMID: 24766769

[41] Cleeland CS. The M. D. Anderson Symptom Inventory User Guide ▪ Version 1 (2016). Available at: https://www.mdanderson.org/documents/Departments-and-Divisions/Symptom-Research/MDASI_userguide.pdf.

[42] Gudala K Ghai B Bansal D. Usefulness of four commonly used neuropathic pain screening questionnaires in patients with chronic low Back pain: a cross-sectional study. Korean J Pain. (2017) 30:51–8. doi: 10.3344/kjp.2017.30.1.51, PMID: 28119771 PMC5256260

[43] Shraim MA Masse-Alarie H Hodges PW. Methods to discriminate between mechanism-based categories of pain experienced in the musculoskeletal system: a systematic review. Pain. (2021) 162:1007–37. doi: 10.1097/j.pain.0000000000002113, PMID: 33136983

[44] Kosek E Clauw D Nijs J Baron R Gilron I Harris RE. Chronic Nociplastic pain affecting the musculoskeletal system: clinical criteria and grading system. Pain. (2021) 162:2629–34. doi: 10.1097/j.pain.0000000000002324, PMID: 33974577

[45] Finnerup NB Haroutounian S Kamerman P Baron R Bennett DLH Bouhassira D. Neuropathic pain: an updated grading system for research and clinical practice. Pain. (2016) 157:1599–606. doi: 10.1097/j.pain.0000000000000492, PMID: 27115670 PMC4949003

[46] Perez C Galvez R Huelbes S Insausti J Bouhassira D Diaz S. Validity and reliability of the Spanish version of the Dn4 (Douleur Neuropathique 4 questions) questionnaire for differential diagnosis of pain syndromes associated to a neuropathic or somatic component. Health Qual Life Outcomes. (2007) 5:66. doi: 10.1186/1477-7525-5-66, PMID: 18053212 PMC2217518

[47] Bouhassira D Attal N Alchaar H Boureau F Brochet B Bruxelle J. Comparison of pain syndromes associated with nervous or somatic lesions and development of a new neuropathic pain diagnostic questionnaire (Dn4). Pain. (2005) 114:29–36. doi: 10.1016/j.pain.2004.12.010 15733628

[48] Mugler JP 3rd Brookeman JR. Three-dimensional magnetization-prepared rapid gradient-Echo imaging (3d Mp rage). Magn Reson Med. (1990) 15:152–7. doi: 10.1002/mrm.1910150117 2374495

[49] Weerasekera A Morrissey E Kim M Saha A Lin Y Alshelh Z. Thalamic Neurometabolite alterations in patients with knee osteoarthritis before and after Total knee replacement. Pain. (2021) 162:2014–23. doi: 10.1097/j.pain.0000000000002198, PMID: 33470749 PMC8205967

[50] Hong D Rohani Rankouhi S Thielen JW van Asten JJA Norris DG. A comparison of Slaser and Mega-Slaser using simultaneous interleaved Acquisition for Measuring Gaba in the human brain at 7t. PLoS One. (2019) 14:e0223702. doi: 10.1371/journal.pone.0223702, PMID: 31603925 PMC6788718

[51] Edden RA Puts NA Barker PB. Macromolecule-suppressed Gaba-edited magnetic resonance spectroscopy at 3t. Magn Reson Med. (2012) 68:657–61. doi: 10.1002/mrm.24391, PMID: 22777748 PMC3459680

[52] Dou W Speck O Benner T Kaufmann J Li M Zhong K. Automatic voxel positioning for Mrs at 7 T. MAGMA. (2015) 28:259–70. doi: 10.1007/s10334-014-0469-9, PMID: 25408107

[53] Ashburner J Barnes G Chen C-C Daunizeau J Flandin G Friston K. Spm12 manual. London: University College London (UCL). (2021). Available at: https://www.researchgate.net/publication/355544981_SPM12_Manual#fullTextFileContent. (Accessed July 22, 2024).

[54] Friston K. Statistical parametric mapping (SPM). SPM12 ed. (2014). Available at: https://www.fil.ion.ucl.ac.uk/spm/

[55] Deelchand D. MRspa: magnetic resonance signal processing and analysis. 1.5th ed (2018) Available at: https://www.cmrr.umn.edu/downloads/mrspa/.

[56] The MathWorks Inc. Matlab Version: 9.13.0 (R2022b). Natick, Massachusetts: The MathWorks Inc. (2022). Available at: https://www.mathworks.com.

[57] Provencher S. Lcmodel. 6.3rd ed. Automatic quantification of *in vivo* proton MR spectra. Available at: http://s-provencher.com/lcmodel.shtml (2021).

[58] Deelchand DK Auerbach EJ Marjanska M. Apparent diffusion coefficients of the five major metabolites measured in the human brain *in vivo* at 3t. Magn Reson Med. (2018) 79:2896–901. doi: 10.1002/mrm.26969, PMID: 29044690 PMC5843522

[59] Fundaun J Kolski M Baskozos G Dilley A Sterling M Schmid AB. Nerve pathology and neuropathic pain after whiplash injury: a systematic review and Meta-analysis. Pain. (2022) 163:e789–811. doi: 10.1097/j.pain.0000000000002509, PMID: 35050963 PMC7612893

[60] Sterling M. Physiotherapy management of whiplash-associated disorders (wad). J Physiother. (2014) 60:5–12. doi: 10.1016/j.jphys.2013.12.004 24856935

[61] Kumbhare DA Elzibak AH Noseworthy MD. Evaluation of chronic pain using magnetic resonance (Mr) neuroimaging approaches: what the clinician needs to know. Clin J Pain. (2017) 33:281–90. doi: 10.1097/AJP.0000000000000415, PMID: 27518493

[62] Seminowicz DA Moayedi M. The dorsolateral prefrontal cortex in acute and chronic pain. J Pain. (2017) 18:1027–35. doi: 10.1016/j.jpain.2017.03.008, PMID: 28400293 PMC5581265

[63] Mills EP Keay KA Henderson LA. Brainstem pain-modulation circuitry and its plasticity in neuropathic pain: insights from human brain imaging investigations. Front Pain Res. (2021) 2:705345. doi: 10.3389/fpain.2021.705345, PMID: 35295481 PMC8915745

[64] Tracey I. Imaging Pain. Br J Anaesth. (2008) 101:32–9. doi: 10.1093/bja/aen102, PMID: 18556697

[65] Malfliet A Coppieters I Van Wilgen P Kregel J De Pauw R Dolphens M. Brain changes associated with cognitive and emotional factors in chronic pain: a systematic review. Eur J Pain. (2017) 21:769–86. doi: 10.1002/ejp.1003, PMID: 28146315

[66] Lin A Andronesi O Bogner W Choi IY Coello E Cudalbu C. Minimum reporting standards for *in vivo* magnetic resonance spectroscopy (Mrsinmrs): experts' consensus recommendations. NMR Biomed. (2021) 34:e4484. doi: 10.1002/nbm.4484, PMID: 33559967 PMC8647919

[67] Boissoneault J Sevel L Letzen J Robinson M Staud R. Biomarkers for musculoskeletal pain conditions: use of brain imaging and machine learning. Curr Rheumatol Rep. (2017) 19:5. doi: 10.1007/s11926-017-0629-9, PMID: 28144827 PMC6875750

[68] Aggarwal K Manso Jimeno M Ravi KS Gonzalez G Geethanath S. Developing and deploying deep learning models in brain magnetic resonance imaging: a review. NMR Biomed. (2023) 36:e5014. doi: 10.1002/nbm.5014, PMID: 37539775

[69] Zhao D Grist JT Rose HEL Davies NP Wilson M MacPherson L. Metabolite selection for machine learning in childhood brain tumour classification. NMR Biomed. (2022) 35:e4673. doi: 10.1002/nbm.4673, PMID: 35088473

[70] Di Costanzo A Trojsi F Tosetti M Schirmer T Lechner SM Popolizio T. Proton Mr spectroscopy of the brain at 3 T: an update. Eur Radiol. (2007) 17:1651–62. doi: 10.1007/s00330-006-0546-1, PMID: 17235536

[71] Jansen JF Backes WH Nicolay K Kooi ME. 1h Mr spectroscopy of the brain: absolute quantification of metabolites. Radiology. (2006) 240:318–32. doi: 10.1148/radiol.2402050314 16864664

[72] Cruz-Almeida Y Porges E. Additional considerations for studying brain metabolite levels across pain conditions using proton magnetic resonance spectroscopy. NeuroImage. (2021) 224:117392. doi: 10.1016/j.neuroimage.2020.117392, PMID: 32971265

[73] Chang L Ernst T Poland RE Jenden DJ. *In vivo* proton magnetic resonance spectroscopy of the normal aging human brain. Life Sci. (1996) 58:2049–56. doi: 10.1016/0024-3205(96)00197-x 8637436

[74] Bliss TV Collingridge GL Kaang BK Zhuo M. Synaptic plasticity in the anterior cingulate cortex in acute and chronic pain. Nat Rev Neurosci. (2016) 17:485–96. doi: 10.1038/nrn.2016.68, PMID: 27307118

[75] Benarroch EE. What is the role of the cingulate cortex in pain? Neurology. (2020) 95:729–32. doi: 10.1212/WNL.0000000000010712 33077671

[76] Zhuo M. Molecular mechanisms of pain in the anterior cingulate cortex. J Neurosci Res. (2006) 84:927–33. doi: 10.1002/jnr.21003 16862566

[77] Hart RP Wade JB Martelli MF. Cognitive impairment in patients with chronic pain: the significance of stress. Curr Pain Headache Rep. (2003) 7:116–26. doi: 10.1007/s11916-003-0021-5 12628053

[78] Xiao X Ding M Zhang YQ. Role of the anterior cingulate cortex in translational pain research. Neurosci Bull. (2021) 37:405–22. doi: 10.1007/s12264-020-00615-2, PMID: 33566301 PMC7954910

[79] Pereira V Goudet C. Emerging trends in pain modulation by metabotropic glutamate receptors. Front Mol Neurosci. (2018) 11:464. doi: 10.3389/fnmol.2018.00464 30662395 PMC6328474

[80] Archibald J MacMillan EL Graf C Kozlowski P Laule C Kramer JLK. Metabolite activity in the anterior cingulate cortex during a painful stimulus using functional Mrs. Sci Rep. (2020) 10:19218. doi: 10.1038/s41598-020-76263-3, PMID: 33154474 PMC7645766

[81] Kameda T Fukui S Tominaga R Sekiguchi M Iwashita N Ito K. Brain metabolite changes in the anterior cingulate cortex of chronic low Back pain patients and correlations between metabolites and psychological state. Clin J Pain. (2018) 34:657–63. doi: 10.1097/AJP.0000000000000583, PMID: 29271797

[82] Ito T Tanaka-Mizuno S Iwashita N Tooyama I Shiino A Miura K. Proton magnetic resonance spectroscopy assessment of metabolite status of the anterior cingulate cortex in chronic pain patients and healthy controls. J Pain Res. (2017) 10:287–93. doi: 10.2147/JPR.S123403, PMID: 28203104 PMC5293371

[83] Shiers S Price TJ. Molecular, circuit, and anatomical changes in the prefrontal cortex in chronic pain. Pain. (2020) 161:1726–9. doi: 10.1097/j.pain.0000000000001897 32701833 PMC7575617

[84] Ong WY Stohler CS Herr DR. Role of the prefrontal cortex in pain processing. Mol Neurobiol. (2019) 56:1137–66. doi: 10.1007/s12035-018-1130-9 29876878 PMC6400876

[85] Loggia ML Berna C Kim J Cahalan CM Martel MO Gollub RL. The lateral prefrontal cortex mediates the Hyperalgesic effects of negative cognitions in chronic pain patients. J Pain. (2015) 16:692–9. doi: 10.1016/j.jpain.2015.04.003, PMID: 25937162 PMC4522376

[86] Bell T Lindner M Langdon A Mullins PG Christakou A. Regional striatal cholinergic involvement in human behavioral flexibility. J Neurosci. (2019) 39:5740–9. doi: 10.1523/JNEUROSCI.2110-18.2019, PMID: 31109959 PMC6636079

[87] Jung C Ichesco E Ratai EM Gonzalez RG Burdo T Loggia ML. Magnetic resonance imaging of Neuroinflammation in chronic pain: a role for Astrogliosis? Pain. (2020) 161:1555–64. doi: 10.1097/j.pain.0000000000001815, PMID: 31990749 PMC7305954

[88] Lentz MR Kim WK Kim H Soulas C Lee V Venna N. Alterations in brain metabolism during the first year of Hiv infection. J Neurovirol. (2011) 17:220–9. doi: 10.1007/s13365-011-0030-9, PMID: 21494901 PMC3753682

[89] Ferrier J Bayet-Robert M Dalmann R El Guerrab A Aissouni Y Graveron-Demilly D. Cholinergic neurotransmission in the posterior insular cortex is altered in preclinical models of neuropathic pain: key role of muscarinic M2 receptors in donepezil-induced Antinociception. J Neurosci. (2015) 35:16418–30. doi: 10.1523/JNEUROSCI.1537-15.2015, PMID: 26674867 PMC4679823

[90] Velazquez R Ferreira E Knowles S Fux C Rodin A Winslow W. Lifelong choline supplementation ameliorates Alzheimer's disease pathology and associated cognitive deficits by attenuating microglia activation. Aging Cell. (2019) 18:e13037. doi: 10.1111/acel.13037, PMID: 31560162 PMC6826123

[91] Castillo M Kwock L Mukherji SK. Clinical applications of proton Mr spectroscopy. AJNR Am J Neuroradiol. (1996) 17:1–15. PMID: 8770242 PMC8337957

[92] Nordengen K Heuser C Rinholm JE Matalon R Gundersen V. Localisation of N-Acetylaspartate in oligodendrocytes/myelin. Brain Struct Funct. (2015) 220:899–917. doi: 10.1007/s00429-013-0691-7, PMID: 24379086

[93] Grachev ID Apkarian AV. Chemical heterogeneity of the living human brain: a proton Mr spectroscopy study on the effects of sex, age, and brain region. NeuroImage. (2000) 11:554–63. doi: 10.1006/nimg.2000.0557 10806041

[94] Roalf DR Sydnor VJ Woods M Wolk DA Scott JC Reddy R. A quantitative meta-analysis of brain glutamate metabolites in aging. Neurobiol Aging. (2020) 95:240–9. doi: 10.1016/j.neurobiolaging.2020.07.015, PMID: 32866885 PMC7609608

[95] Lind A Boraxbekk CJ Petersen ET Paulson OB Siebner HR Marsman A. Regional Myo-inositol, Creatine, and choline levels are higher at older age and scale negatively with visuospatial working memory: a cross-sectional proton Mr spectroscopy study at 7 tesla on Normal cognitive ageing. J Neurosci. (2020) 40:8149–59. doi: 10.1523/JNEUROSCI.2883-19.2020, PMID: 32994337 PMC7574655

[96] Harrell F. Regression modeling Startegies: with applications to linear Odels, logistic and ordinal regression and survival analysis. 2nd ed. New York: Springer Series in Statistics (SSS). (2015). Available at: https://www.springer.com/series/692.

[97] Peek AL Leaver AM Foster S Oeltzschner G Puts NA Galloway G. Increased Gaba+ in people with migraine, headache, and pain conditions-a potential marker of pain. J Pain. (2021) 22:1631–45. doi: 10.1016/j.jpain.2021.06.005, PMID: 34182103 PMC8935361

[98] Freitag P Greenlee MW Wachter K Ettlin TM Radue EW. Fmri response during visual motion stimulation in patients with late whiplash syndrome. Neurorehabil Neural Repair. (2001) 15:31–7. doi: 10.1177/154596830101500105, PMID: 11527277

[99] Wang WE Ho RLM Ribeiro-Dasilva MC Fillingim RB Coombes SA. Chronic jaw pain attenuates neural oscillations during motor-evoked pain. Brain Res. (2020) 1748:147085. doi: 10.1016/j.brainres.2020.147085, PMID: 32898506

[100] Brand A Richter-Landsberg C Leibfritz D. Multinuclear Nmr studies on the energy metabolism of glial and neuronal cells. Dev Neurosci. (1993) 15:289–98. doi: 10.1159/000111347, PMID: 7805581

