# Supplementary material for: Chronic neuropathic pain components in whiplash-associated disorders correlate with metabolite concentrations in the anterior cingulate and dorsolateral prefrontal cortex: a consensus-driven MRS re-examination
Source: Front Med (Lausanne). 2024 Jul 29;11:1404939. doi: 10.3389/fmed.2024.1404939 (PMC11328873; doi:10.3389/fmed.2024.1404939)
Supplement: Supplementary file 1 [file Table_1.docx]

**Supplementary Tables**

**Supplementary Table 1.** MRSinMRS checklist for reporting single voxel cerebral ^1^H-MRS imaging parameters including hardware, acquisition, data analysis and quality control.

| 1. **Hardware** | |
| --- | --- |
| 1. Field strength [T] | 3T |
| 1. Manufacturer | Siemens |
| 1. Model (software version if available): | SIEMENS MAGNETOM TrioTim syngo MR B19 |
| 1. RF coils: nuclei (transmit/receive), number of channels, type, body part | 32-channel Head coil receiver |
| 1. Additional hardware: | N/A |
| 1. **Acquisition** | |
| 1. Pulse sequence: | MEGA-semi-LASER SVS (mslaser) |
| 1. Volume of interest (VOI) and locations, nominal VOI size **[**mm^3^**]** | ACC 🡪 35x35x10 mm^3^  OCCIPITAL 🡪 20x20x20 mm^3^  DLPFC 🡪 20x20x40 mm^3^ |
| 1. Repetition time (TR) [ms] | 3000 ms |
| 1. Echo Time (TE) [ms] | 85.36 ms |
| 1. Total number of excitations or acquisitions per spectrum | 128 averages (64 off, 64 on) |
| 1. Additional sequence parametres |  |
| - Spectral width in Hz | 2000 Hz |
| - Number of spectral points | 2048 points |
| - Frequency offsets | -1.7ppm |
| 1. Water suppression method: | VAPOR with optional embedded outer  volume suppression (OVS) pulses to suppress water and to improve the localization of the volume of interest (VOI). |
| 1. Shimming method, reference peak, and thresholds for “acceptance of shim” chosen | Siemens shim “Brain” shim mode (System 3D-GRE) |
| 1. Triggering or motion correction method (respiratory, peripheral, cardiac triggering, incl. device used and delays) | N/A |
| 1. **Data analysis methods and outputs** | |
| 1. Analysis software | SPM12 for segment the anatomical images from each participant to determine the fractions of GM, WM and CSF in VOIs.  MRspa for spectral process (corrected for eddy current effect, frequency and phase shifts).  LCModel for quantification of metabolites concentrations and ratios. |
| 1. Processing steps deviating from quoted reference or product | Custom simulated basis set |
| 1. Output measure (concentration (mM) concentration, concentration [mM], ratio), processing steps deviating from quoted reference or product | Concentration of metabolites (mM) and ratios to total creatine |
| 1. Quantification references and assumptions, fitting model assumptions: | Each spectrum was calibrated by setting creatine peak (3.03ppm) for amplitude phase in the frequency correction. |
| 1. **Data quality** | |
| 1. Reported variables | |
| 1. SNR | **OCC:** 22.53 ± 6.11; FWHM: 0.048 ± 0.027 ppm.  **ACC:** 26.08 ± 4.37; FWHM: 0.035 ± 0.028 ppm.  **DLPFC:** 28.45 ± 5.87; FWHM: 0.039 ± 0.010 ppm as reported by LCmodel.  None eliminated |
| 1. Linewidth (Hz) | OCCIP: 6.98 ± 1.57  ACC: 6.67 ± 1.20  DLPFC: 6.78 ± 1.08 |
| 1. Data exclusion criteria | Linewidth > 10 Hz  CRLB > 20% |
| 1. Quality measures of postprocessing model fitting (CRLB, goodness of fit, SD of residual) | CRLB of NAA: 2 ± 0 (2) % (edit off) |
| 1. Sample spectrum | See Figure 1 |

**Supplementary Table 2.** ^1^H-MRS data quality characteristics for the Occipital cortex (OCC), Anterior Cingulate Cortex (ACC), and Left Dorsolateral Prefrontal Cortex (DLPFC) in the non-injured and whiplash injury (WHI) groups.

|  | OCCIP - non-injured | | OCCIP - WHI |
| --- | --- | --- | --- |
|  | |  |  |
| SNR Edit (On) dB | 20.97 ± 4.38 | | 17.86 ± 5.67***** |
| SNR Off dB | 27 (24-29) | | 26 (24-29) |
| Linewidth (LW) Hz | 6.88 ± 1.51 | | 7.08 ± 1.64 |
| FWHM edit (On) ppm | 0.04 (0.04-0.05) | | 0.048 (0.04-0.06) |
| FWHM edit Off ppm | 0.04 (0.04-0.05) | | 0.048 (0.038-0.055) |
| % CSF | 11.96 ± 3.68 | | 12.18 ± 3.35 |
| CRLB%_[tCr]_ | 2 (2-2) | | 2 (2-2) |
| CRLB%_[tCho]_ | 4 (3-4) | | 4 (3-4) |
| CRLB%_[NAA]_ | 1 (1-1) | | 1(1-1)**#** |
| CRLB%_[Glx]_ | 12 (10-12) | | 12 (10-13.25) |
| CRLB%_[Ins]_ | 7 (6.75-8.00) | | 7 (6.75-8.00) |
| CRLB%_[GABA]_ | 18 (17.00-22.25) | | 21 (18.00-24.00) |
| CRLB%_[Glu]_ | 27 (21.00-33.50) | | 28 (22.00-43.75) |
|  | **ACC - non-injured** | | **ACC - WHI** |
|  | | - | 37.60 (30.43-49.57) |
| SNR Edit (On) | 26.62 ± 4.43 | | 23.93 ± 4.85* |
| SNR Off | 28.07 ± 3.37 | | 25.69 ± 3.83* |
| Linewidth (LW) Hz | 6.35± 0.9 | | 6.99 ± 1.39* |
| FWHM edit (On) ppm | 0.028 (0.027-0.032) | | 0.032 (0.028-0.042)# |
| FWHM Off ppm | 0.024 (0.024-0.032) | | 0.040 (0.027-0.048)## |
| % CSF | 12.4 ± 3.35 | | 12.58 ± 3.08 |
| CRLB%_[tCr]_ | 2 (2-2) | | 2(2-2) |
| CRLB%_[tCho]_ | 2 (2-2) | | 2(2-2) |
| CRLB%_[NAA]_ | 1 (1-1) | | 1(1-1) |
| CRLB%_[Glx]_ | 9 (9-10) | | 9 (9-11) |
| CRLB%_[Ins]_ | 6 (6-7) | | 6 (6-7) |
| CRLB%_[GABA]_ | 16 (15.00-19.00) | | 17 (15.00 - 19.25) |
| CRLB%_[Glu]_ | 13 (12-15) | | 15 (13.75 – 20.00)## |
|  | **DLPFC - non-injured** | | **DLPFC - WHI** |
|  | |  |  |
| SNR Edited (On) | 30.15 ± 6.89 | | 26.18 ± 6.77 |
| SNR Off | 30.31 ± 3.33 | | 28.00 ± 5.16 |
| Linewidth (LW) Hz | 6.79 (6.42-7.23) | | 7.52 (6.77-7.79) |
| FWHM edited (On) ppm | 0.032 (0.028-0.042) | | 0.040 (0.032-0.048) |
| FWHM Off ppm | 0.032 (0.032-0.040) | | 0.040 (0.032-0.042) |
| % CSF | 5.9 ± 3.98 | | 5.81 ± 3.16 |
| CRLB%_[tCr]_ | 2 (2-2) | | 2 (2-2) |
| CRLB%_[tCho]_ | 2 (2-2) | | - |
| CRLB%_[NAA]_ | 1 (1-1) | | 1 (1-1) |
| CRLB%_[Glx]_ | 9 (8-10) | | 9 (8.00-9.25) |
| CRLB%_[Ins]_ | 5 (5-5.5) | | 5 (5-6) |
| CRLB%_[GABA]_ | 18.42 ± 4.10 | | 22.53 ± 6.50 |
| CRLB%_[Glu]_ | 15.5 (14.5-17.0) | | 17 (15.5-23.5) |

Data shown as mean (± SD) or median scores (25th to 75th percentiles). * Student t-test (p<0.05). # (Mann-Whitney test, p<0.05). ## (Mann-Whitney test, p<0.01).
